# Supervised Exercise Immediately After Bariatric Surgery: the Study Protocol of the EFIBAR Randomized Controlled Trial

**DOI:** 10.1007/s11695-021-05559-8

**Published:** 2021-07-15

**Authors:** Enrique G. Artero, Manuel Ferrez-Márquez, María José Torrente-Sánchez, Elena Martínez-Rosales, Alejandro Carretero-Ruiz, Alba Hernández-Martínez, Laura López-Sánchez, Alba Esteban-Simón, Andrea Romero del Rey, Manuel Alcaraz-Ibáñez, Manuel A. Rodríguez-Pérez, Emilio Villa-González, Yaira Barranco-Ruiz, Sonia Martínez-Forte, Carlos Castillo, Carlos Gómez Navarro, Jesús Aceituno Cubero, Raúl Reyes Parrilla, José A. Aparicio Gómez, Pedro Femia, Ana M. Fernández-Alonso, Alberto Soriano-Maldonado

**Affiliations:** 1 Department of Education, Faculty of Education Sciences; SPORT Research Group (CTS-1024), CERNEP Research Centre, University of Almería, Almería, Spain; 2 General and Bariatric Surgery Unit, Torrecárdenas University Hospital, Almería, Spain; 3 Hospital Mediterráneo, Almería, Spain; 4 Pediatric Unit, Torrecárdenas University Hospital, Almería, Spain; 5 Department of Education and Health Research Centre, University of Almería, Almería, Spain; 6 PROFITH Promoting Fitness and Health through Physical Activity Research Group, Department of Physical and Sports Education, Faculty of Education and Sport Sciences, Sport and Health University Research Institute (iMUDS), University of Granada, Melilla, Spain; 7 Obstetrics and Gynecology Unit, Torrecárdenas University Hospital, Almería, Spain; 8 Department of Economics and Business, SPORT Research Group (CTS-1024), CERNEP Research Centre, University of Almería, Almería, Spain; 9 Unit of Cardiology, Torrecárdenas University Hospital, Almería, Spain; 10 Department of Statistics and Operations Research, Faculty of Medicine, University of Granada, Granada, Spain

**Keywords:** Obesity, Bariatric surgery, Exercise, Randomized controlled trial (RCT), Protocol

## Abstract

**Background:**

Previous studies have investigated weight loss caused by exercise following bariatric surgery. However, in most cases, the training program is poorly reported; the exercise type, volume, and intensity are briefly mentioned; and the sample size, selection criteria, and follow-up time vary greatly across studies.

**Purpose:**

The EFIBAR study aims to investigate over 1 year the effects of a 16-week supervised exercise program, initiated immediately after bariatric surgery, on weight loss (primary outcome), body composition, cardiometabolic risk, physical fitness, and quality of life in patients with severe/extreme obesity.

**Material and Methods:**

The EFIBAR study is a parallel-group, superiority, randomized controlled trial (RCT), comprising 80 surgery patients. Half of the participants, randomly selected, perform a 16-week supervised exercise program, including both strength and aerobic training, starting immediately after the surgery (7–14 days). For each participant, all primary and secondary outcomes are measured at three different time points: (i) before the surgery, (ii) after the intervention (≈4 months), and (iii) 1 year after the surgery.

**Conclusion:**

The EFIBAR study will provide new insights into the multidimensional benefits of exercise in adults with severe/extreme obesity following bariatric surgery.

**Trial Registration:**

EFIBAR randomized controlled trial was prospectively registered at Clinicaltrials.gov (NCT03497546) on April 13, 2018.

**Figure:**
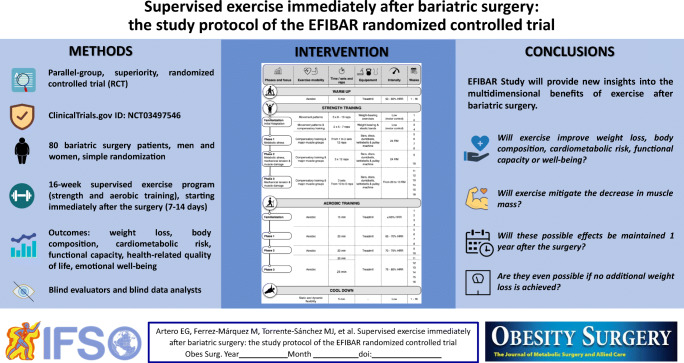
Graphical abstract

## Introduction

Bariatric surgery is an effective treatment option for reducing weight in people with extreme obesity [1], on average losing around 12% of total body weight in the first 6 months and up to 45% over 3 years [2]. Physical activity (PA) and exercise reduce comorbidities related to obesity, such as asthma and sleep problems [3], insulin resistance, hypertension, and hypercholesterolemia [4]. Furthermore, exercise plays an important role in the amount of weight regained following weight loss and helps to reduce weight progressively [5]. Given the increasing number of people with extreme obesity [6], the proven short- and long-term effectiveness of bariatric surgery [7], and the possibilities presented by exercise in relation to the maintenance and improvement of risk factors [8], PA could help those patients who suffer weight regain after bariatric surgery [9].

Previous studies have investigated weight loss caused by exercise following bariatric surgery, generally suggesting the suitability of PA for improving bariatric surgery outcomes [10–12]. However, in many cases, the training program is poorly reported [13]; the exercise type, volume, and intensity are briefly mentioned; and the sample size, selection criteria, and follow-up time vary greatly across studies [14]. The EFIBAR randomized controlled trial (*Ejercicio FÍsico tras cirugía BARiátrica*, Physical Exercise after Bariatric Surgery) aims to investigate over 1 year the effects of a 16-week supervised exercise program, initiated immediately after bariatric surgery, on weight loss, body composition, cardiometabolic risk, physical fitness, and quality of life in patients with severe/extreme obesity. Even if additional weight loss is not achieved [14], exercise following bariatric surgery may help to maintain lean body mass, improve cardiovascular health and psychological well-being, and increase adherence to PA, among other benefits [9]. The results of this study may help to assess the effectiveness of exercise as a therapeutic complement to bariatric surgery.

## Materials and Methods

### Study Design, Protocol Registration, and Reporting

The EFIBAR study is a parallel-group, superiority, randomized controlled trial (RCT) registered at www.clinicaltrials.gov (NCT03497546) on April 13, 2018, before the enrollment of participants began (May 1, 2018). The design of the study, as well as this protocol manuscript, follows the SPIRIT reporting guideline [15].

### Recruitment and Eligibility Criteria

#### Recruitment

The recruitment process takes place through the Bariatric Surgery Units of Torrecárdenas University Hospital and Hospital Mediterráneo, both located in the city of Almería (Spain). In both centers, the surgical team includes surgeons accredited by the Spanish Society for Obesity and Metabolic Surgery (SECO), performing the interventions under the same criteria.

#### Eligibility Criteria

The participants’ inclusion and exclusion criteria are listed in Table 1.

**Table 1:** Eligibility criteria

Inclusion criteria	Exclusion criteria
Age 18-60 yrs.	Severe psychiatric or neurological disorders such as schizophrenia, epilepsy, Alzheimer’s, Parkinson’s, personality disorders, eating behavior disorders, untreated depression, or suicidal tendencies
BMI ≥ 40 kg/m^2^ (or ≥ 35 kg/m^2^ with comorbid conditions)	
Acceptable surgical risk (defined by the approval of an anesthetist)	
Obesity maintained for over 5 years	Adrenal or thyroid pathology that might cause obesity
Failure of previous treatments	
Not presenting contraindications for supervised physical exercise	Uncontrolled addiction to alcohol or drugs
To reside in the city of Almería (or willingness/predisposition to attend the training sessions 3 times a week over 16 weeks)	

### Sample Size

Assuming an alpha error of 0.05 and a power of 80%, a total of 66 patients (n=33 patients per group) are needed to detect an effect (between group difference) of at least 0.7 standard deviations [16] in the % of total weight loss (% TWL). Anticipating a potential follow-up loss of up to 20%, a total of 80 patients will be recruited (i.e., 40 per group). Based on previous literature and our surgical team’s experience, 60–80% of the recruited participants are expected to be women.

### Randomization

Participants are randomly assigned to the experimental (EG) or control group (CG). A member of our research team (statistician), not involved in any other section of the study, created a computer-generated sequence of simple randomization [17]. The sequence is concealed using sealed and opaque envelopes numbered sequentially. Only patients who meet all the selection criteria, sign an informed consent, and finally undergo the surgery receive a randomization number, corresponding to the allocation sequence previously generated. After the surgery, at medical discharge, a nurse opens the envelope in front of the patient, in the absence of any blinded member of the research team.

### Blinding

Because of the type of intervention (exercise program), it is not possible to mask either the patients or the surgeon, given that participants inevitably reveal the group allocation during postoperative follow-up consultations. However, researchers responsible for evaluation and data analysis are blinded to the group assignment (*blind evaluator* and *blind data analyst*, respectively). In addition, personal trainers in charge of implementing the exercise program are not part of the assessment team.

### Data collection

#### Assessment Protocol

**Figure 1:**
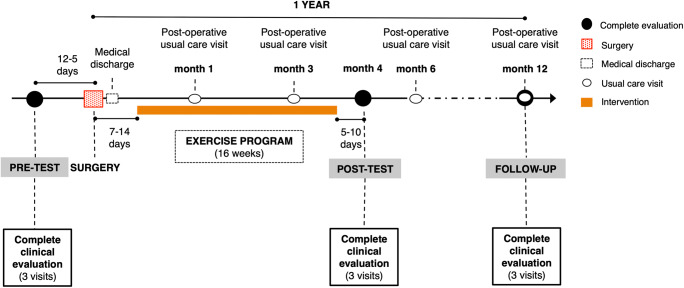
Data collection diagram.

Each participant receives three complete clinical assessments over the study: (1) pre-surgery evaluation, 5–12 days before the surgery; (2) post-intervention evaluation, 5–10 days after completing the intervention phase (i.e., approximately 4 months after the surgery); and (3) follow-up evaluation, 1 year after the surgery (Figure 1).

#### Primary Outcome

The percentage of total weight loss (%TWL) has been recommended for expressing weight loss after surgery [18, 19]: % TWL = [(pre-surgery weight – post-surgery weight)/(pre-surgery weight)] × 100. In the EFIBAR study, weight is measured using a bioimpedance device (InBody 270, Biospace Co., USA).

#### Secondary Outcomes

1. *Body composition*. Electric bioimpedance (InBody 270, Biospace Co., USA; Lookin’Body 120 software) is used to measure fat mass (FM, kg), fat-free mass (FFM, kg), and percentage of body fat (%BF). All participants are asked to urinate before the assessment, and to fast for at least 2h. Height is measured using a portable system (SECA 213, Hamburg, Germany) with the patient shoeless in a standing position. Body mass index (BMI) is calculated as weight (kg) divided by height squared (m^2^). Waist and hip circumferences are measured in cm with an anthropometric tape, following standardized guidelines [20].
2. *Cardiovascular diseases risk biomarkers*. Fasting blood samples are collected and processed by the *Sistema Sanitario Público de Andalucía* (SSPA) Biobank, according to standard procedures: (2.1) lipid profile: total cholesterol (mg/dl), HDL-cholesterol (mg/dl), LDL-cholesterol (mg/dl), and triglycerides (mg/dl); (2.2) glycemic control: glucose (mg/dl), insulin (μUI/ml), glycated hemoglobin HbA1c (%), and insulin resistance (homeostasis model assessment, (HOMA-I), [21]); (2.3) chronic inflammation: high-sensitivity C-reactive protein [hs-CRP, (mg/dl)], tumor necrosis factor alpha [TNF-alpha (pg/ml)] and interleukin 6 [IL-6, (pg/ml)]; (2.4) liver metabolism enzymes: alanine aminotransferase (ALT, U/L), aspartate aminotransferase (AST, U/L), and alkaline phosphatase (ALP, U/L).
3. *Blood pressure and arterial stiffness*: brachial and central blood pressures (mm/Hg), resting heart rate (HR, bpm) and arterial stiffness [pulse wave velocity, PWV (m/seg)] are measured using a Mobil-O-Graph® oscillometry-based pulse analysis system (IEM GmbH, Stolberg, Germany) [22, 23]. Three readings are recorded with the participant resting quietly in a sitting position for 10 min, the cuff placed on the upper left arm around the brachial artery, and the palm facing upwards.
4. *Heart-rate variability (HRV)*. Measurements take place in the morning between 8:30 AM and 11:30 AM in a quiet, temperature-controlled room (22–24°C). Participants are requested not to exercise or to drink caffeinated and/or alcoholic beverages for 24 h prior to the examination and to fast for at least 3 h. Participants are instructed to breathe normally and not to talk or fidget while measurements are being taken. Duration of RR intervals is recorded using a Polar V800 telemetry heart-rate monitor (Polar Electro Oy, Kempele, Finland). The electrode belt is dampened and placed just below the chest muscles. Heart rhythm is recorded over 10 min at a sampling frequency of 1000 Hz with the patient seated. Kubios Premium software (v.3.2, Heart Rate Analysis, University of Eastern Finland) is used to process the raw HRV data [24, 25].
5. *Health-related fitness (HRF) and physical activity (PA)*. (5.1) Cardiorespiratory fitness is assessed using a maximal treadmill test following the Bruce protocol [26, 27]. Maximal oxygen uptake (VO_2max_) in METs is estimated based on time to exhaustion. Rate of perceived exertion (RPE, Borg_1–10 scale_), maximum HR (bpm), and recovery HR (1, 2, and 3 min afterwards, bpm) are also recorded. (5.2) Hand-grip strength (kg) is measured using a digital hand dynamometer (T.K.K. 5401 Grip-D; Takei, Tokyo, Japan). The patient stands with the shoulder slightly abducted (~10°), the elbow extended, and the forearm and wrist in a neutral position. Each participant performs the test three times (alternately with both hands) allowing a 1-min rest between measurements. Grip span is adapted to hand size differently for men and women [28]. (5.3) Lower body muscular strength: 30-s chair stand test. This measures the number of full stand-ups from a seated position that participants can complete in 30 s with arms folded across chest [29]. (5.4) Upper body (shoulder) flexibility: back scratch test (cm). With one hand reaching over the shoulder and one up the middle of the back, the number of centimeters between the extended middle fingers is recorded (plus or minus). The best of two trials is used for analysis [29]. (5.5) Objectively measured PA and sedentary time are assessed by accelerometry (ActiGraph; ActiLife version 6.11.7 software) [30, 31]. Patients wear a triaxial accelerometer on the hip for 24 h (except for bathing or water activities) that records acceleration in all three movement axes (in min/day) of PA performed over a total of 7 days. The information is collected over a 60-s period at a sampling rate of 30 Hz [30]. The criterion for considering a record valid is to fulfill a minimum of 10 h a day for 4 days [30].
6. *Patient-reported outcomes.* (6.1) Health-related quality of life is assessed using the Spanish version of the 36-item Short-Form Health Survey (SF-36v2) [32], together with the European Quality of Life-5 Dimensions (EQ-5D) [33]. (6.2) Symptomatology and function of hip/knee osteoarthritis is measured using the WOMAC (Western Ontario and McMaster Universities) Osteoarthritis Index [34, 35]. (6.3) Depression, anxiety and stress are assessed using the Depression, Anxiety and Stress Scale short form (DASS-21) [36]. (6.4) Emotional, psychological, and social well-being are measured using the Mental Health Continuum-Short Form (MHC-SF) [37].
7. *Cost-effectiveness analysis (CEA) and cost-utility analysis (CUA)*. The costs of the two treatments under consideration (bariatric surgery and bariatric surgery plus exercise) will be investigated from a dual perspective: the National Health Service and the patient. The first approach will consider the surgery, exercise program, prescription medication, sick leave, postoperative complications, and readmission rates. The patient-centered approach will additionally include the time and effort requiring each treatment, as well as any possible dietary and informal care costs. Effectiveness will be evaluated in terms of changes observed in the main clinical outcomes, i.e., weight loss and cardiometabolic risk. On the other hand, utility will be evaluated as variation in quality of life experienced by participants (quality-adjusted life years, QALY), using SF-36 and EQ-5D questionnaires. All primary and secondary outcomes are shown in Table 2.

**Table 2:** Summary of the time-point measurements of the study outcomes

Measurement	Pre-surgery	Month 1	Month 3	Post-intervention (month 4)	Month 6	Month 9	Follow-up (month 12)
Primary outcome							
Weight loss	x	x	x	x	x	x	x
Secondary outcomes							
Body composition^a^	x			x			x
CVD risk biomarkers^b^	x			x			x
Central and brachial BP, resting HR, PWV	x			x			x
HRV	x			x			x
Health-related fitness and physical activity ^c^	x			x			x
Health-related quality of life (SF-36v2)	x			x			x
Health-related quality of life (EQ-5D)	x	x	x	x	x	x	x
Symptomatology and function of hip/knee osteoarthritis (WOMAC)	x			x			x
Depression, anxiety, and stress (DASS-21)	x			x			x
Emotional, psychological, and social well-being (MHC-SF)	x			x			x

*BP* blood pressure (mm/Hg), *CVD* cardiovascular diseases, *DASS-21* Depression, Anxiety and Stress Scale short form, *EQ-5D* European Quality of Life-5 Dimensions, *HR* heart rate (bpm), *HRV* heart rate variability, *MHC-SF* Mental Health Continuum-Short Form, *PWV* pulse wave velocity (m/seg), *SF-36v2* 36-item Short-Form Health Survey, *WOMAC* Western Ontario and McMaster Universities Osteoarthritis Index

^a^Body fat (%), fat mass (kg), fat-free mass (kg), body mass index (BMI; kg/m^2^), waist and hip circumferences (cm)

^b^Total cholesterol (mg/dl), HDL-cholesterol (mg/dl), LDL-cholesterol (mg/dl), triglycerides (mg/dl), glucose (mg/dl), insulin (μUI/ml), glycated hemoglobin HbA1c (%), insulin resistance [homeostasis model assessment, (HOMA-I)], high-sensitivity C-reactive protein [hs-CRP, (mg/dl)], tumor necrosis factor [TNF-alpha (pg/ml)], interleukin 6 [IL-6, (pg/ml)], alanine aminotransferase (ALT, U/L), aspartate aminotransferase (AST, U/L) and alkaline phosphatase (ALP, U/L)

^c^Cardiorespiratory fitness (VO_2max_, METs), hand-grip strength (kg), 30-s chair-stand test (number of repetitions), back scratch test (cm), and objectively measured physical activity (accelerometry)

#### Control Variables and Other Parameters to Be Recorded

Different variables are recorded that may influence the study results even though they are not part of the intervention: (1) sex, age, educational level, marital status, occupational status, and income level; (2) personal history of obesity (obesity duration), cardiovascular disease, hypertension, obstructive sleep apnea (OSA), type 2 diabetes, and medication use; (3) surgical technique (see below); (4.1) adherence to Mediterranean diet [38, 39]; and (4.2) usual intake estimates of food groups, energy, and nutrients using a self-administered semi-quantitative food-frequency questionnaire (FFQ) [40] and 24-h recalls.

### Intervention

#### Bariatric Surgery

Three different techniques are used in this study, all of them carried out using laparoscopic surgery: sleeve gastrectomy (SG), gastric bypass (GB), and one anastomosis gastric bypass (OAGB). The technique used for each participant is decided by a multidisciplinary team of endocrinologists, nutritionists, psychologists, and surgeons, based on patient’s BMI, comorbidities, and age, among other considerations. The distribution of the three procedures (SG, GB, and OAGB) is expected to be the same in the two groups (control vs. experimental).

#### Control Group (CG, Usual Care)

Participants in both groups receive the postoperative follow-up routinely prescribed in both medical centers, according to international standards [41]. During the first 4 weeks, all patients maintain a semi-liquid diet. Medical consultations are scheduled at months 1, 3, 6, 9, and 12 after the surgery, with special attention to nutritional status. Written counseling is given, focused on the benefits of a healthy diet and regular physical activity.

#### Experimental Group (EG, Usual Care + Supervised Exercise)

**Figure 2.**
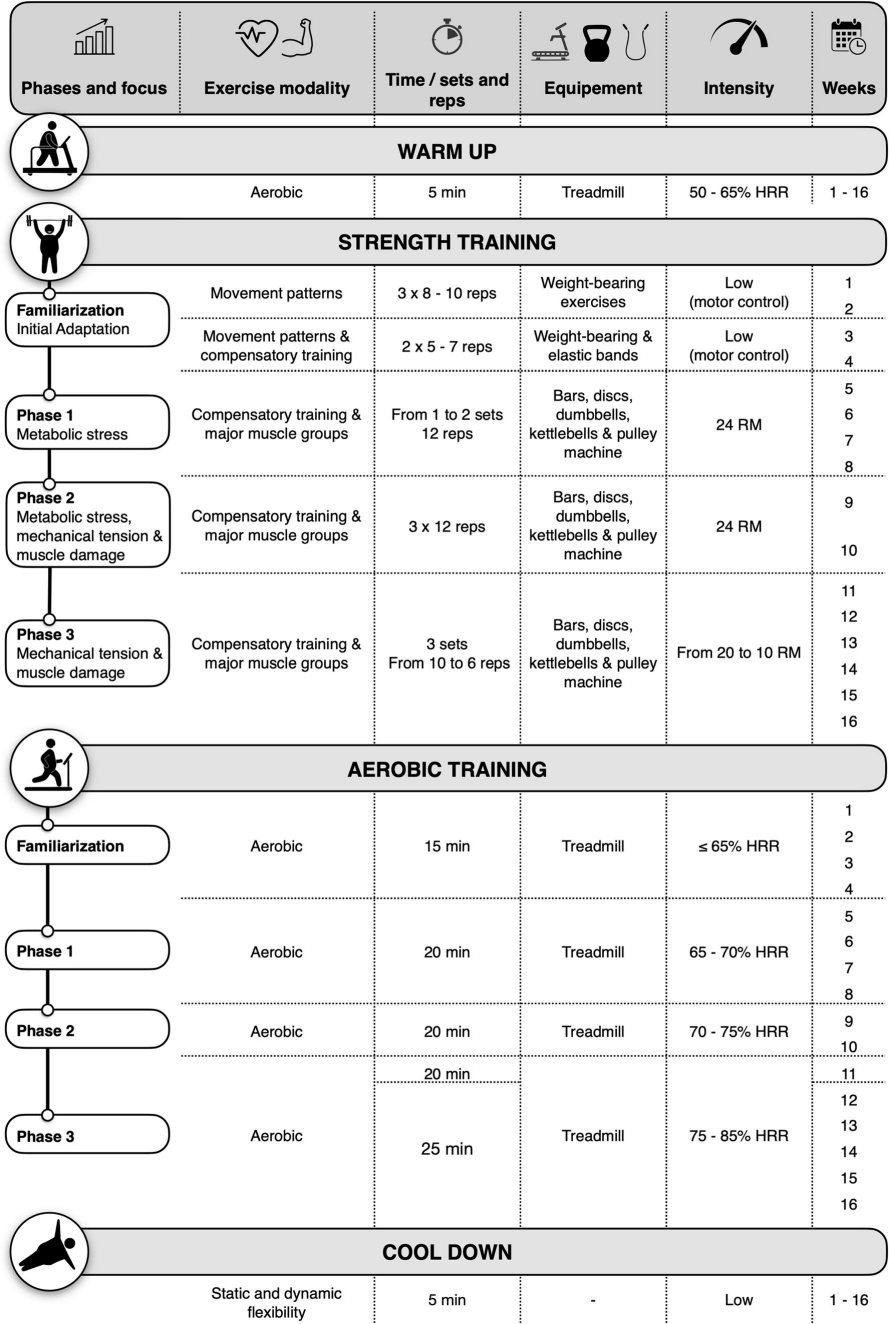
Overview of the EFIBAR training program (for more details, see [42]). HRR: heart-rate reserve, min: minutes, reps: repetitions, RM: repetition maximum.

The EFIBAR training program has been published elsewhere [42] and was designed and reported following the CERT guidelines [43]. The program begins 7–14 days after surgery, exercising 3 times a week over 16 weeks (first 4 weeks of familiarization), using 60-min sessions that includes (1) warm up [light aerobic activity at 50–65% of HR reserve (HRR)], (2) compensatory training (i.e., core stability and stabilizer muscle exercises), (3) strength training (whole body exercises progressing from 1 to 3 sets, from 12 to 6 repetitions per set, from 24 to 10 repetitions maximum (RM) (≈50 to 75% of 1RM), (4) aerobic training (on a treadmill, progressing from 15 to 25 min, from 65 to 85% of HRR), and (5) cool down (static and dynamic stretching exercises) [42] (Figure 2). Maximum HR is recorded during the maximum treadmill test, while resting HR is taken from the HRV assessment, both included in the pre-surgery evaluations. Resting HR is re-assessed twice throughout the program (sessions 12th and 36th), and the corresponding aerobic training zones are properly re-calculated. Two members of the research team, with BSc degrees in Sport Sciences and at least 2 years of experience as personal trainers, are in charge of implementing the exercise program. This is performed individually and supervised, with a 1:1 ratio unless participants report schedule unavailability (allowing, in this case, two patients with the same personal trainer) [42]. Attendance, punctuality, training HR, rate of perceived exertion (RPE and ONMI), mood, adverse events, and extra physical activity, among others, are recorded daily. Performing at least 80% of all planned training sessions will be considered a successful attendance rate. *WhatsApp* messages and videos are sent to participants every Friday and every month, respectively, to encourage adherence throughout the intervention [42].

### Statistical Analysis

To test whether the randomization has worked effectively, baseline values will be compared for potential clinically important differences. The difference between groups in the change (post-pre) in % TWL (primary outcome) and all secondary outcomes will be analyzed using the generalized linear model, with the post-pre difference as the dependent variable, and the group (EG or CG), time (16 weeks or 12 months), and their interaction, as the fixed effects. The effect size (95% confidence interval) and statistical significance will be reported for the effects of group (between-subjects), time (intra-subject), and the group × time interaction. The primary analysis will follow per-protocol analysis (only including participants with available data who attend at least 80% of the exercise sessions). Sensitivity analyses will be performed using the intention-to-treat (ITT) principle. For that purpose, any missing data will be replaced through multiple imputations using regression models with age, type of surgical technique, and baseline values as predictors. The analyses will be adjusted for baseline values plus relevant confounding variables. Stata v.13.1 or higher (StataCorp LP, College Station, TX) will be used to conduct the analyses, and the statistical significance will be set at P <.05.

## Discussion

Obesity is certainly one of the most serious and prevalent health problems currently challenging our society. In less than 5 years (by 2025), obesity is expected to reach a global prevalence of 20%, with almost half of that corresponding to severe/extreme obesity [44]. The burden that this group of patients poses to the health and economic systems is remarkable [45], as a direct result of the treatment costs (usually long-term) but also because of the interruption of their contribution to the production system. Any strategy that can improve their prognosis and allow them to return to society with recovered functional capacity will have a huge impact, not just on them but also on their communities, leading to increased quality of life, productivity, and economic growth.

Our study will primarily investigate whether weight loss 1 year after surgery is greater among those who followed the exercise program. More interestingly, we will be able to test whether this exercise program can mitigate the decrease in muscle mass that normally accompanies bariatric surgery [46]. In addition, this study includes multiple health-related outcomes other than weight loss and body composition, such as lipid and glycemic profiles, chronic inflammation, liver biomarkers, physical fitness, PA, and health-related quality of life. Our experience in previous PA intervention studies [47–52] leads us to believe that the aforementioned health outcomes are more sensitive to exercise and can even be improved in the absence of weight loss [53, 54]. A significant improvement in any of these parameters with no additional weight loss would be a plausible—and very interesting—finding.

## Conclusion

The EFIBAR study will provide new insights into the multidimensional benefits of exercise in adults with severe/extreme obesity following bariatric surgery. Using a wide variety of clinical, psychological, and socio-economic indicators, this study will compare the combination of supervised exercise and usual care with usual care alone. We hypothesize that a 16-week exercise program using strength and aerobic training, implemented immediately after bariatric surgery, will improve weight loss, body composition, cardiometabolic risk, fitness levels, PA, health-related quality of life, and psychological well-being, to a greater extent than the usual care. By using cost-effectiveness and cost-utility analyses, we will also test whether the designed intervention is not only feasible and safe, but also effective and useful from an economic standpoint.
